# Supplementary material for: Criterion-related validity of Bedriddenness Rank with other established objective scales of ADLs, and Cognitive Function Score with those of cognitive impairment, both are easy-to-use official Japanese scales: A prospective observational study
Source: PLoS One. 2022 Nov 10;17(11):e0277540. doi: 10.1371/journal.pone.0277540 (PMC9648766; doi:10.1371/journal.pone.0277540)
Supplement: S1 Appendix — (PDF) [file pone.0277540.s001.pdf]

**Supporting Information file**

**S1, Appendix. Characteristics of Yuai-Kai Foundation and Oda Hospital**

The hospital contains 11 departments with 111 beds for acute care. The departments are: Internal Medicine, General Medicine, General Surgery, Cardiovascular Surgery, Neurosurgery, Otorhinolaryngology, Plastic Surgery, Dermatology, Radiology, Anesthesiology, and Rehabilitation. The facility has no Orthopedic Surgery department. The hospital is located in the city of Kashima, in Saga Prefecture in southern Japan. It covers an approximate population of 90,000 people, and treats approximately 3,100 inpatients each year, with a mean length of stay of 12.1 days.
