# Supplementary material for: Criterion-related validity of Bedriddenness Rank with other established objective scales of ADLs, and Cognitive Function Score with those of cognitive impairment, both are easy-to-use official Japanese scales: A prospective observational study
Source: PLoS One. 2022 Nov 10;17(11):e0277540. doi: 10.1371/journal.pone.0277540 (PMC9648766; doi:10.1371/journal.pone.0277540)
Supplement: S1 Table — Table A. The categories of Bedriddenness Ranks. Table B. The categories of Cognitive Function Scores. (PDF) [file pone.0277540.s002.pdf]

S1, Table A. The categories of Bedriddenness Ranks.

Category	Criteria	Subcategory	Characteristics
**J independence /autonomy**	Slight disorders with being almost independent in daily living and can outing.	**J1**	Use public transport.
		**J2**	Go out only neighborhood.
**A house-bound**	Almost independent at home without the ability of going out alone.	**A1**	Spend most time away from the bed and sometimes go out with some assistance.
		**A2**	Spend most time in bed and rarely go out even with some assistance.
**B chair-bound**	Dependent even at home and spend almost time in bed in the daytime with the ability of keeping sitting position alone.	**B1**	Move to wheelchair alone, eat and excrete away from bed.
		**B2**	Move to wheelchair with help.
**C bed-bound**	Bedridden and dependent of excretion, eating and gowning.	**C1**	Roll over alone.
		**C2**	Roll over with help.

S1, Table B. The categories of Cognitive Function Scores.

Category	Criteria	Subcategory	Characteristics
**1**	Almost independent in daily living only with slight cognitive impairment.		
**2**	Independent with slight difficulty in daily living or communication under careful overseeing.	**2a**	Applicable only outside.
		**2b**	Applicable even at home.
**3**	Dependent in daily living or communication.	**3a**	Applicable mainly daytime.
		**3b**	Applicable mainly nighttime.
**4**	Dependent in daily living or communication with requiring constant care.		
**M**	Severe psychological symptoms, troubled behaviors or severe physical disorders requiring specialized medical service.		
