# Supplementary material for: Criterion-related validity of Bedriddenness Rank with other established objective scales of ADLs, and Cognitive Function Score with those of cognitive impairment, both are easy-to-use official Japanese scales: A prospective observational study
Source: PLoS One. 2022 Nov 10;17(11):e0277540. doi: 10.1371/journal.pone.0277540 (PMC9648766; doi:10.1371/journal.pone.0277540)
Supplement: S2 Table — (PDF) [file pone.0277540.s003.pdf]

S2, Table. The breakdown of the diseases leading to patients' hospitalization according to the International Statistical Classification of Diseases and Related Health Problems 10th revision (ICD-10).

ICD-10 category	n (%)
A: Certain infectious and parasitic diseases	91 (3.0)
B: Certain infectious and parasitic diseases	127 (4.2)
C: Neoplasms	332 (11.1)
D: Diseases of the blood and blood-forming organs and certain disorders involving the immune mechanism	229 (7.6)
E: Endocrine, nutritional and metabolic diseases	118 (3.9)
F: Mental, Behavioral and Neurodevelopmental disorders	17 (0.6)
G: Diseases of the nervous system	64 (2.1)
H: Diseases of the eye, ear, and mastoid process	147 (4.9)
I: Diseases of the circulatory system	517 (17.2)
J: Diseases of the respiratory system	504 (16.8)
K: Diseases of the digestive system	341 (11.4)
L: Diseases of the skin and subcutaneous tissue	153 (5.1)
M: Diseases of the musculoskeletal system and connective tissue	27 (0.9)
N: Diseases of the genitourinary system	99 (3.3)
Q: Congenital malformations, deformations and chromosomal abnormalities	13 (0.4)
R: Symptoms, signs and abnormal clinical and laboratory findings, not elsewhere classified	17 (0.6)
S: Injury, poisoning and certain other consequences of external causes	89 (3.0)

T: Injury, poisoning and certain other consequences of external causes	77 (2.6)
Z: Factors influencing health status and contact with health services	1 (0.0)
Unknown	40 (1.3)
