# Supplementary material for: Criterion-related validity of Bedriddenness Rank with other established objective scales of ADLs, and Cognitive Function Score with those of cognitive impairment, both are easy-to-use official Japanese scales: A prospective observational study
Source: PLoS One. 2022 Nov 10;17(11):e0277540. doi: 10.1371/journal.pone.0277540 (PMC9648766; doi:10.1371/journal.pone.0277540)
Supplement: Attachment — Submitted filename: 2022-09-15_Response.docx [file pone.0277540.s004.docx]

When submitting your revision, we need you to address these additional requirements.

1. Please ensure that your manuscript meets PLOS ONE's style requirements, including those for file naming. The PLOS ONE style templates can be found at

https://journals.plos.org/plosone/s/file?id=wjVg/PLOSOne_formatting_sample_main_body.pdf and

<https://journals.plos.org/plosone/s/file?id=ba62/PLOSOne_formatting_sample_title_authors_affiliations.pdf>

**Response:**

We revised the manuscript in accord with the template provided by PLOS ONE.

2. Please provide additional details regarding participant consent. In the ethics statement in the Methods and online submission information, please ensure that you have specified what type you obtained (for instance, written or verbal, and if verbal, how it was documented and witnessed). If your study included minors, state whether you obtained consent from parents or guardians. If the need for consent was waived by the ethics committee, please include this information.

**Response:**

We confirmed that all of the required details were included in our manuscript.

3. Thank you for stating the following in the Competing Interests section:

Masaki Tago is supported by grants from Japan Society for the Promotion of Science, JSPS KAKENHI Grant Number JP18K17322 and JP21H03166. The sponsor of the study had no role in the study design, data collection, analysis, or preparation of the manuscript.

Please confirm that this does not alter your adherence to all PLOS ONE policies on sharing data and materials, by including the following statement: "This does not alter our adherence to PLOS ONE policies on sharing data and materials.” (as detailed online in our guide for authors http://journals.plos.org/plosone/s/competing-interests). If there are restrictions on sharing of data and/or materials, please state these. Please note that we cannot proceed with consideration of your article until this information has been declared.

Please respond by return email with your amended Competing Interests Statement and we will change the online submission form on your behalf.

**Response:**

We changed the corresponding sentences in the Competing Interests Statement as instructed (as described below), which we made sure to include in the cover letter and our return e-mail.

Masaki Tago is supported by grants from Japan Society for the Promotion of Science, JSPS KAKENHI Grant Number JP18K17322 and JP21H03166. The sponsor of the study had no role in the study design, data collection, analysis, or preparation of the manuscript. This does not alter our adherence to PLOS ONE policies on sharing data and materials.

4. Your ethics statement should only appear in the Methods section of your manuscript. If your ethics statement is written in any section besides the Methods, please delete it from any other section.

**Response:**

We deleted unnecessary ethics statements in sections other than the Methods.

Reviewers' comments:

Reviewer's Responses to Questions

Comments to the Author

1. Is the manuscript technically sound, and do the data support the conclusions?

The manuscript must describe a technically sound piece of scientific research with data that supports the conclusions. Experiments must have been conducted rigorously, with appropriate controls, replication, and sample sizes. The conclusions must be drawn appropriately based on the data presented.

Reviewer #1: Yes

Reviewer #2: No

2. Has the statistical analysis been performed appropriately and rigorously?

Reviewer #1: Yes

Reviewer #2: No

3. Have the authors made all data underlying the findings in their manuscript fully available?

The PLOS Data policy requires authors to make all data underlying the findings described in their manuscript fully available without restriction, with rare exception (please refer to the Data Availability Statement in the manuscript PDF file). The data should be provided as part of the manuscript or its supporting information, or deposited to a public repository. For example, in addition to summary statistics, the data points behind means, medians and variance measures should be available. If there are restrictions on publicly sharing data—e.g. participant privacy or use of data from a third party—those must be specified.

Reviewer #1: Yes

Reviewer #2: Yes

4. Is the manuscript presented in an intelligible fashion and written in standard English?

PLOS ONE does not copyedit accepted manuscripts, so the language in submitted articles must be clear, correct, and unambiguous. Any typographical or grammatical errors should be corrected at revision, so please note any specific errors here.

Reviewer #1: Yes

Reviewer #2: No

5. Review Comments to the Author

**Response to the comments of Reviewer 1**

Reviewer #1: The proposed work seeks to provide further validation of the Bedriddenness Rank (BR) as a measure of ADL limitations, previously validated in a smaller study, as well as to assess validity of the Cognitive Function Score (CFS) as a measure of cognitive impairment. While I commend the authors for a well-written manuscript, I would encourage them to consider the following suggestions to further improve their work:

**Response:**

We appreciate Reviewer 1’s assessment of our manuscript as “well-written.” Our responses to Reviewer 1’s comments are provided below.

1. Provide a better description of BR and CFS, particularly what each category (e.g., J1, J2 for BR and I, IIa etc. for CFS) indicates. Although this information is available from prior publications of the group, access to it within the manuscript would save the reader time they would otherwise have to spend searching for it.

Response:

In accord with Reviewer 1’s recommendation, we have described the details of the categories of Bedriddenness Ranks (BR) and Cognitive Function Scores (CFS) in S1, Table, to make the information more accessible to readers. Additionally, we added a sentence to lines 159 to 160 on page 12, as follows.

Line 159–160,

The detailed categories of BR and CFS were shown in S1, Tables A and B.

2. Lines 169-170 suggest that the CFS M category was defined in this study as ‘severe psychiatric symptoms, problematic behavior, or serious physical illness requiring medical care’, while in the previous validation study (Tago M et al., 2021; doi: https://doi.org/10.1186/s12877-0021-02108-x) the same category was defined as ‘Does the patient have delirium or self-inflicted harm?’. A better description of CFS (see point #1) would help clarify this confusion.

**Response:**

We apologize for the confusion regarding the definition of category M of CFS in the previous version of our manuscript. The question, “Does the patient have delirium or self-inflicted harm?”, which was included in our previous research, was not the precise definition of category M, but a simplified criterion to facilitate the work of making a judgment in a busy medical setting. We would like to emphasize that the precise definition of category M is “severe psychiatric symptoms, problematic behavior, or serious physical illness requiring specialized medical care.” as reported in our original manuscript. The definition of category M is clearly described in S1, Table, which we added to address a previous comment by Reviewer 1.

3. How long does CFS take to administer? The authors imply that some of the other available assessment tools (e.g., MMSE) are lengthy and require certain equipment to administer. However, at only 7.4 minutes average time and requiring paper and a few objects (for naming) that can be anything that is available to the rater, MMSE is generally considered a quick test that does not require special training or equipment and is widely used in the clinical setting due in part to its ease of administration. I would downplay this as a main rationale for assessing validity of CFS as a cognitive assessment.

**Response:**

We appreciate Reviewer 1’s comment. CFS is an extremely easy-to-use tool, which requires less than 2 minutes to perform with a maximum of five steps, as we already mentioned in the background section. However, we did not measure the precise length of time necessary for the evaluation of CFS. Therefore, we added a sentence to clarify that we did not evaluate the time necessary for the evaluation of CFS in this study in the Limitations section in lines 306 to 308 on page 22, as described below.

Line 306-308,

In addition, the time required for the evaluation of BR or CFS, the simplicity of the procedures, and the validity of the assessment method of ABC-DS, were not evaluated in this study.

However, we do not agree that Mini-Mental State Examination (MMSE) is a sufficiently simple and quick test. In busy clinical settings in Japan, a required duration of 7.4 minutes is too long to be useful for diagnosing dementia for a single outpatient in a clinic overloaded with large numbers of older patients. In addition, in current and actual clinical settings in Japan, medical staff with experience conducting the MMSE are often unavailable, mainly for economic reasons. Moreover, the MMSE requires the preparation of materials such as paper, pens, and evaluation sheets. Thus, we believe that the MMSE involves a substantially more complicated procedure than CFS.

4. For the CFS-ABC-DS analysis, consider doing a sensitivity analysis focused on the 951 patients who underwent MMSE testing. This group was more cognitively impaired (median MMSE score = 15, which indicates moderate dementia) compared to the sample of 3,003 patients with data on ABC-DS (median ABC-DS score = 117, which is equivalent to a global CDR of 0/0.5 indicating normal cognition/very mild cognitive impairment (Mori T et al., 2018; doi: 10.1159/000486956). This is likely due to the inclusion of patients with normal CFS scores in the ‘ABC-DS’ sample while they were excluded from the ‘MMSE’ sample. Assessing correlation with the two ‘gold-standard’ tests in the same sample would improve comparability of the two CFS validation analyses. Since MMSE was not done in patients with normal CFS scores, how about removing them for the ABC-DS as well? Alternatively, the CFS-MMSE correlation analysis could be repeated including the 29 patients who were excluded due to protocol deviation (CFS normal despite MMSE assessed), although I would consider doing this as a second choice given the small number of patients with normal CFS scores that would be added to the ‘MMSE’ sample.

**Response:**

Following the recommendation of Reviewer 1, we calculated Spearman’s correlation coefficient between CFS and the ABC Dementia Scale (ABC-DS), among the patients for whom MMSE was assessed because their CFS was abnormal. The correlation coefficient showed a strong negative correlation between the two scales with a correlation coefficient of r = −0.698 (p < 0.001), which we described in lines 216 to 219 on page 17, as described below.

Line 216-219,

Additionally, Spearman’s rank correlation coefficients of CFS with ABC-DS in the group of patients for whom MMSE was evaluated because their CFS was abnormal, revealed a strong negative correlation, with a correlation coefficient of r = −0.698 (p < 0.001).

5. I encourage the authors to consider validating CFS against the cognitive domain of ABC-DS in addition to the global score. That would provide a more direct assessment of CFS’s validity as a cognitive impairment measure and would be more consistent with the validation against MMSE.

**Response:**

We believe that it is acceptable to compare the total score of ABC-DS with MMSE, because its parallel validity with MMSE has been confirmed in previous studies. In addition, it was impossible to evaluate the point suggested by Reviewer 1 because of the unfortunate lack of results for each domain (A: ADL, B: BPSD, and C: Cognitive function). We appreciate the reviewer’s understanding.

6. Finally, could the authors comment on how ABC-DS was administered? Was the rating done by nurses/medical clerks? If yes, how valid would it be since the original scale was developed using input from caregivers who assist the patients 3 or more days a week (see the Mori article)?

**Response:**

In the current study, attending nurses evaluated and recorded ABC-DS by interviewing caregivers who accompanied the patient. We added a sentence to clarify this point in lines 154 to 155 on page 11, as described below.

Lines 154-155,

ABC-DS was evaluated and recorded by an attending nurse by interviewing a caregiver who accompanied the patient.

In addition, as Reviewer 1 mentioned, we did not confirm whether the caregivers assisted the patients for 3 or more days a week. Therefore, we added a sentence to describe this point in the Limitations section in lines 306 to 308 on page 22, as described below.

Line 306-308,

In addition, the time required for the evaluation of BR or CFS, the simplicity of the procedures, and the validity of the assessment method of ABC-DS, were not evaluated in this study.

**Response to the comments of Reviewer 2**

Reviewer #2: First of all, I would like to apologize for the delay in reviewing the manuscript. Generally, I try to do it as fast as possible but I was unable to because of an unexpectedly busy schedule in the last month.

**Response:**

We thank Reviewer 2 for reviewing our manuscript. Our responses to Reviewer 2’s comments are provided below.

1. The current study investigates the criterion-related validity of Bedriddenness Rank (BR) and Cognitive Function Score (CFS) in subjects aged 20 years and older who were hospitalized in a suburban city in Japan. The major concern is that the subjects in the current study were probably hospitalized for a wide range of disorders that may differently affect ADLs and cognitive function. Meanwhile, it is recommended to investigate the validity and reliability of instruments/questionnaires in specific populations. In addition, no information about the reason for the hospitalization of the participants is provided in the manuscript.

**Response:**

We appreciate Reviewer 2’s helpful comment. We added ICD-10 classification of the diseases that led to patients’ hospitalization as S2, Table, except for data for age, gender, and length of hospital stay as inpatient information, which we had already described in our original manuscript. We added a sentence to describe this point in lines 193 to 195 on page 14, as described below.

Lines 193-195,

A breakdown of the diseases leading to patients’ hospitalization according to the International Statistical Classification of Diseases and Related Health Problems 10th revision [23] is shown in S2, Table.

We would like to emphasize that the inter-rater reliability of the BR and CFS were already confirmed in a previous study, although we did not evaluate the validity and reliability of the various instruments in the current study, as mentioned by Reviewer 2. In addition, we do not believe that evaluation of the validity and reliability of the Barthel index (BI), Katz index (KI), or MMSE are necessary, because they have been widely used in numerous studies as well-established objective instruments. However, because we failed to validate the ABC-DS assessment methods, as already mentioned in our response to Reviewer 1’s comment, we added a sentence to describe this point in the Limitations section in lines 306 to 308 on page 22, as described below.

Lines 306-308,

In addition, the time required for the evaluation of BR or CFS, the simplicity of the procedures, and the validity of the assessment method of ABC-DS, were not evaluated in this study.

2. Further, although the main focus of the introduction is the aging population in Japan as a super-aged society and the need to assess ADLs and cognitive function in hospitalized elderly subjects using brief and easy-to-use instruments, the authors recruited hospitalized subjects aged 20 years or older.

**Response:**

First, we already demonstrated the correlations between BR and BI, BR and KI, CFS and BI, and CFS and KI for older patients, respectively, in our previous study. The current study involved adult patients that were 20 years of age or older. However, the median age of the study population was 77 years, because this study was conducted in the hospital in which most of the admitted patients were older people, causing no logical inconsistency in our manuscript.

Second, following Reviewer 1’s recommendation, we also calculated Spearman’s correlation coefficients between CFS and the ABC Dementia Scale (ABC-DS), among patients for whom MMSE was assessed because their CFS was abnormal. As a result, the correlation coefficient showed a strong negative correlation between those two scales with a correlation coefficient of r = −0.698 (p < 0.001), which meant that we succeeded in the validation regarding CFS in aged patients with dementia. We added a sentence to demonstrate this point at lines 216 to 219 on page 17, as described below. We hope that this resolves Reviewer 2’s concern.

Line 216-219,

Additionally, Spearman’s rank correlation coefficients of CFS with ABC-DS in the group of patients for whom MMSE was evaluated because their CFS was abnormal, revealed a strong negative correlation, with a correlation coefficient of r = −0.698 (p < 0.001).

3. Another important issue is the low necessity to do such a study. As mentioned in the introduction, the criterion-related validity of the BR and CFS is re-evaluated in the current study. Further, as mentioned by the authors, CFS is usually used to measure the limitations of ADLs caused by dementia and it does not assess cognitive function. So, merely the significant correlation of CFS with MMSE or ABC-DS does not justify the use of CFS for assessing cognitive impairment instead of MMSE or ABC-DS.

**Response:**

We respectfully disagree with Reviewer 2’s comment on this point. The fact that the present study revealed a significant correlation between CFS and MMSE or ABC-DS indicates that CFS had the power to predict MMSE and ABC-DS scores. Furthermore, we propose that CFS has the potential to be used as an objective dementia scale considering the results of previous research and the present study, because the inter-rater reliability of CFS has already been established in a previous study [17]. We described this point in lines 129 to 132 on page 10, as described below.

Lines 129-132,

Although CFS is usually used to measure limitations of ADLs caused by dementia, it could potentially be used for assessing cognitive impairment, if significant correlations with the MMSE or ABC-DS were demonstrated.

In addition, we stated in our manuscript that CFS showed significant criterion-related validity with well-established but complicated and time-consuming cognitive impairment scales such as MMSE or ABC-DS, including in the Conclusions section. We did not comment that CFS should be used as a dementia scale instead of these scales because of its significant correlations, in the Discussion or in the Conclusions. We appreciate Reviewer 2’s understanding.
