# Supplementary material for: Criterion-related validity of Bedriddenness Rank with other established objective scales of ADLs, and Cognitive Function Score with those of cognitive impairment, both are easy-to-use official Japanese scales: A prospective observational study
Source: PLoS One. 2022 Nov 10;17(11):e0277540. doi: 10.1371/journal.pone.0277540 (PMC9648766; doi:10.1371/journal.pone.0277540)
Supplement: Attachment — Submitted filename: Response to Reviewers.docx [file pone.0277540.s005.docx]

We thank the editor for reviewing our manuscript. We responded to the editor's comments as follows.

1. In the Methods section, consider incorporating the response provided to Reviewer 1 comment #5. This will clarify, for the reader, why the C domain of the ABC-DS, which would be a more direct measure of cognitive function, was not used as a validation measure in this study.

**Response:**

As we had explained in the previous revision, only the total score of the ABC-DS is currently available, because of imperfect records by nurses. Therefore, we added the explanation to the Methods section at Line 152-153 on Page 10, as described below.

At Line 152-153 on Page 10

Only the data of total score of ABC-DS was available.

2. In the Results section, provide some context for why the sensitivity analysis was conducted.

**Response:**

We explained why we conducted the sensitivity analysis in the Results section at Line 215-220 on Page 16, as described below.

At Line 215-220 on Page 16

Additionally, we conducted a sensitivity analysis focusing on the group of patients**,** for whom MMSE was evaluated because their CFS was abnormal**,** to improve comparability of the validation analyses between CFS and MMSE, and CFS and ABC-DS. As a result, Spearman’s rank correlation coefficient of CFS with those patients was −0.698 (p < 0.001).

3. In addition, and related to point #2 above, an interpretation of the sensitivity analysis results should be provided in the Discussion section. As it stands right now, neither the rationale for the sensitivity analysis nor the interpretation of its results are provided, potentially leaving the reader wondering why the analysis was carried out and what it accomplished.

**Response:**

We provided the interpretation of the sensitivity analysis results in the Discussion section at Line 274-277 on Page 19, as described below.

At Line 274-277 on Page 19

Furthermore, Spearman’s rank correlation coefficients of CFS with ABC-DS in the group of patients for whom MMSE was evaluated because their CFS was abnormal, revealed a strong negative correlation, with a correlation coefficient of r = −0.698 (p < 0.001).

4. The authors should more thoroughly address the first issue raised by Reviewer 2, providing not just a breakdown of diseases leading to hospitalization in the sample but also their potential impact, if any, on study findings.

**Response:**

We conducted a sensitivity analysis by ICD-10 category, as the editor indicated. For categories with sufficient sample size (BR: C, I, J, and K, CFS: I and J), the correlation coefficients between BR or CFS and other scales were similar to those of the overall cohort in this study. On the other hand, there regrettably were some categories (BR: F and H, CFS: B, C, D, R, and S) with insufficient sample sizes resulting in low correlation coefficients, which we considered unreliable. Furthermore, because the validation of correlations between BR or CFS and other scales for each disease category is quite different from the main objective of this study, and the analysis results were unreliable, we decided not to add those results in the main text. In addition, we had already described this point in the Limitation section at Line 305-308 on Page 21, “Thus, the current study may have been affected by population bias caused by the limited number of hospitals, clinical departments, kinds of diseases, and unique characteristics of the region to which the hospital belonged.” We hope the editor understands our intention.

5. In addition, revise Table S2 to provide a description of each presented ICD-10 code category (e.g., E codes (Endocrine, nutritional and metabolic diseases)).

**Response:**

We added the specific categories of ICD-10 to S2, Table, following the editor's suggestion.
